# Meta-analysis of primary open versus closed cannulation strategy for totally implantable venous access port implantation

**DOI:** 10.1007/s00423-020-02057-w

**Published:** 2021-01-09

**Authors:** Ulla Klaiber, Pascal Probst, Matthes Hackbusch, Katrin Jensen, Colette Dörr-Harim, Felix J. Hüttner, Thilo Hackert, Markus K. Diener, Markus W. Büchler, Phillip Knebel

**Affiliations:** 1 Department of General, Visceral and Transplantation Surgery, University of Heidelberg, Im Neuenheimer Feld 420, 69120 Heidelberg, Germany; 2 Study Centre of the German Surgical Society, University of Heidelberg, Heidelberg, Germany; 3 Institute of Medical Biometry and Informatics, University of Heidelberg, Heidelberg, Germany

**Keywords:** Venous access ports (TIVAP), Seldinger technique, *Venae sectio*, Open cut-down, Pneumothorax

## Abstract

**Background:**

There is still no reference standard for the implantation of totally implantable venous access ports (TIVAPs). A recently published multicentre randomised controlled trial (RCT) revealed a significantly greater risk of pneumothorax after closed cannulation than after an open strategy. The aim of this meta-analysis was to provide an update of the available evidence for the safety and effectiveness of primary open versus closed cannulation strategy.

**Methods:**

RCTs comparing outcomes of open cut-down of the cephalic vein and closed cannulation of the subclavian vein were sought systematically in MEDLINE, Web of Science and CENTRAL. The primary outcome was the occurrence of pneumothorax. A beta-binominal model was applied to combine the respective outcomes, and results are presented as odds ratios (OR) with 95% confidence interval (CI).

**Results:**

Six RCTs with a total of 1831 patients were included in final analysis. Meta-analysis showed statistically significant superiority of the open cut-down technique regarding pneumothorax (OR 0.308, 95% CI 0.122 to 0.776), but a statistically significant higher failure of the primary technique for the open cut-down technique than for closed cannulation (OR 2.364, 95% CI 1.051 to 5.315). There were no significant differences between the two procedures regarding other morbidity endpoints.

**Conclusion:**

This meta-analysis shows a general superiority of open cut-down of the cephalic vein over closed cannulation of the subclavian vein regarding the occurrence of pneumothorax. Open cut-down should be the first-line approach for TIVAP implantation. Closed cannulation should be performed with ultrasound as second-line procedure if the open technique fails.

**Systematic review registration:**

PROSPERO CRD42013005180

**Supplementary Information:**

The online version contains supplementary material available at 10.1007/s00423-020-02057-w.

## Introduction

Since the introduction of totally implantable venous access ports (TIVAPs) by Niederhuber *et al.* in 1982 [1], TIVAP implantation has rapidly developed into the most widely preferred treatment option for patients who need a safe and permanent venous access, especially for repeated administration of chemotherapy [2, 3]. Because of the continuing increase in the incidence of oncological diseases and the increasing value of systemic treatment in many malignancies, the number of TIVAP implantations worldwide is likely to grow further [4–6]. Today, the two main approaches for TIVAP placement are [1] closed cannulation—preferably of the subclavian vein—followed by insertion of the catheter in Seldinger technique, and [2] surgical insertion of the catheter into the cephalic vein through an open cut-down technique. The different implantation techniques have been investigated in a large number of non-comparative and comparative studies, including randomised controlled trials (RCTs) [3, 7]. Two meta-analyses published in 2014 and 2016 compared open cut-down of the cephalic vein with closed cannulation of the subclavian vein. Both showed that closed cannulation was superior to open cut-down in terms of primary success rate, while there was no significant difference between the two procedures regarding the occurrence of postoperative complications, especially pneumothorax [7, 8]. The Cochrane meta-analysis stated that the comparison of pneumothorax was restricted due to the low numbers of events. The modified open cut-down (using a guide wire with peel-away sheath through the cephalican vein) could not be evaluated because only one trial was available in 2016. In the meantime, the largest RCT to date PORTAS-3 was published. This trial gives additional data in all of these areas of interest and has shown that the risk of pneumothorax or haemothorax is significantly higher in patients undergoing closed cannulation than in those treated with the primary open strategy [9]. Considering that pneumothorax and haemothorax are potentially life-threatening events which in many cases require further invasive treatment as well as admission to hospital, these complications are highly relevant for both the individual patient and the health-care system [10]. It is good scientific practice to update meta-analyses when new RCTs are published to examine if they have a significant impact on the existing evidence. As the results from this recent RCT including 1205 patients substantially increase the existing body of evidence [9], the aim of the present systematic review and meta-analysis was to provide an update of the available data on the safety and effectiveness of primary open versus closed implantation of TIVAPs.

## Methods

The conduct and results of this study were reported according to the Preferred Reporting Items for Systematic Reviews and Meta-Analyses (PRISMA) Statement [11]. This systematic review was registered prospectively in PROSPERO (registration number: CRD42013005180) and the study protocol was published open access [12]. Funding was granted by the German Federal Ministry of Education and Research (grant number: 01KG1217).

### Data sources and search strategy

A systematic literature search was performed according to the recommendations of the Cochrane Collaboration [13]. Searches were conducted to identify all published and unpublished RCTs referring to the safety and effectiveness of primary open versus closed cannulation for TIVAP implantation. Non-randomised and non-comparative studies investigating at least one of both procedures were also searched to review the existing literature (the ‘Supplementary information section’). Systematic literature searches were conducted in MEDLINE (via PubMed), Embase and The Cochrane Central Register of Controlled Trials (CENTRAL) from the Cochrane Library as described in the study protocol [12]. The last electronic search was carried out on 17 December 2019 [14]. The search was not restricted to specific languages or by the status of the publication. Studies published before 1982 were not considered, as in that year Niederhuber et al. published the first report of TIVAP implantation [1]. In MEDLINE, the related citation function was used to search for additional relevant studies. Additionally, a hand search of the reference lists of relevant articles and related systematic reviews was performed. Experts in the field were asked to supply the latest information on trial results. Furthermore, the registries in ClinicalTrials.gov, Current Controlled Trials, the UMIN Clinical Trials Registry and PROSPERO were searched to identify ongoing and unpublished trials and reviews. Registry data were checked against the data in the final publication. Additionally, for each study included for review, the publication of the study protocol was sought and, if available, checked.

### Study selection

Two investigators independently reviewed all records identified by the abovementioned search methods. Only studies meeting the following eligibility criteria were included: RCTs and non-randomised and non-comparative studies providing data on perioperative and postoperative complications of at least one technique for TIVAP implantation (open cut-down of the cephalic vein and/or closed cannulation of the subclavian vein) predominately due to underlying oncological disease in patients with at least 15 years of age. Case reports, trials investigating TIVAP implantation in children and studies focusing on patients with non-malignant diseases such as cystic fibrosis, sickle cell anaemia, immunodeficiency syndrome and other non-oncological diseases with impaired immune or coagulation system were excluded. If the title and abstract suggested relevance, the full article was assessed for eligibility. Patients undergoing primary puncture of the internal jugular vein were also excluded as this access is not the first choice due to its worse cosmetic result with required long-distance tunnelling of the catheter. Studies in which other percutaneous access sites (basilic, cephalic, femoral etc.) were used, or in which the access site was not specified, were excluded as well to reduce clinical heterogeneity. Any disagreements between the two reviewers were discussed within the working group.

### Data extraction

All predefined data and outcome variables were extracted by two authors independently from the studies included for review. If there was any disagreement between the two reviewers, a third author was consulted. The following items were extracted: title of trial, authors’ names, year of publication, journal, trial period, trial design and sample size. The baseline data extracted were age and sex of participants, underlying disease and surgical procedures. Furthermore, all relevant outcome parameters were extracted. Results from populations based on the intention-to-treat (ITT) principle were collected (i.e. patients were analysed according to the group to which they were randomised). If the results reported in the primary publication were not based on the ITT population, the authors were asked to provide ITT data.

### Outcomes

The primary outcome was the occurrence of pneumothorax. The secondary outcomes were failure of the primary technique, overall morbidity, mortality, dislocation of the catheter or port chamber, TIVAP-associated thrombosis, postoperative bleeding/haematoma, early and late reintervention, early and late malfunction of TIVAPs, TIVAP-associated infection, extravasation, nerve lesion, pinch-off phenomenon, TIVAP occlusion and hospital readmission due to TIVAP problems.

### Assessment of methodological quality

The methodological quality of the included RCTs was assessed by means of version 2 of the Cochrane risk-of-bias tool (RoB 2) [15]. RoB 2 comprises a fixed set of different domains of bias with respect to trial design, trial conduct and trial reporting. For each domain, signalling questions were answered to identify sources of bias with regard to the primary outcome. Based on the answers, an algorithm generated a judgement regarding the risk of bias (‘low’, ‘high’ or ‘some concerns’) arising from each domain.

### Statistical analysis

The odds ratio (OR) with 95% confidence interval (CI) was used as effect measure for all outcomes. If the pooled 95% CI does not include the neutral effect of 1, the results are considered statistically significant. The OR was defined as the odds for the primary open strategy divided by the odds for the closed technique. Therefore, an OR smaller than 1 indicates a significant advantage for the open strategy. A meta-analysis was performed only if more than three RCTs provided data on the respective outcome. Random-effects models were used to combine the effect estimates and their standard errors, as clinical heterogeneity between the included studies was assumed. Meta-analyses were based on ITT data from the primary studies. An available case analysis was conducted as primary analysis, as missing values were not imputed. Due to the known low event rates for some outcomes (especially pneumothorax), in some of the included RCTs, no event was expected to occur in either one or both arms. Therefore, as recommended in this situation, the beta-binomial model was applied [16]. A continuity correction of 0.5 per cell was used only for visualisation of the effects in the forest plots and to examine the statistical heterogeneity by calculating an estimate of the between-studies variance *τ*^2^. Potential publication bias was assessed by visual examination of the funnel plot for the primary outcome (i.e. occurrence of pneumothorax). To evaluate the influence of the missing values for the main outcome, a best-case/worst-case analysis was performed as sensitivity analysis. As a second sensitivity analysis, a pooled OR with its 95% credible interval was derived by fitting a Bayesian random-effects meta-analysis to the data of the outcome pneumothorax [17]. For the treatment effect, a non-informative normal prior distribution (N(0,10^4) and for the between-studies variance, a log-normal prior distribution based on the values for the outcome category ‘surgical/device-related success/failure’ was used for this sensitivity analysis [18]. R version 3.6.1 [19] and the R meta package version 4.9-7 [20], as well as SAS (version 9.4) along with SAS macros, provided in the supportive information by Kuss et al., were used for analysis and visualisation [16]. The second sensitivity analysis was performed with the software jags [21], using the rjags package [22].

## Results

### Study selection

**Fig. 1:**
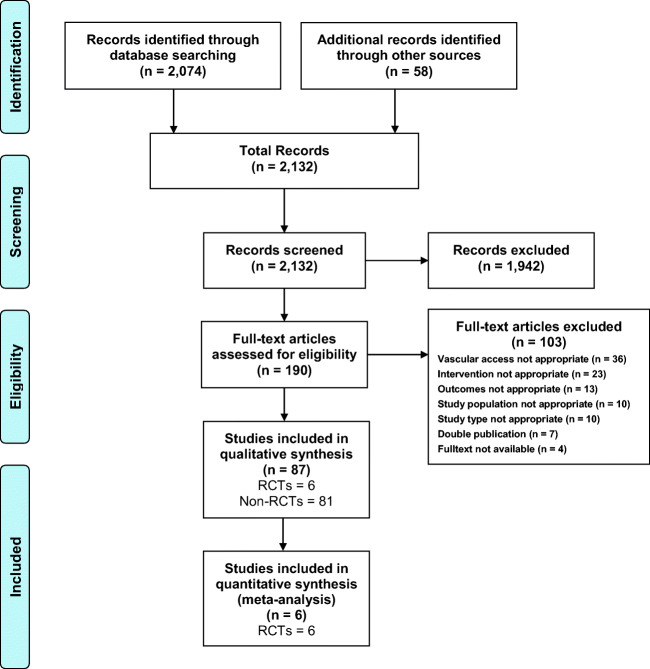
PRISMA flow diagram showing selection of articles for review

The combined search methods yielded a total of 2,132 records (Fig. 1). After screening titles and abstracts, 1942 records were excluded and the full texts of the remaining 190 articles were assessed for eligibility. An additional 103 articles were then excluded because they did not fulfil the inclusion criteria. The remaining 87 articles included six RCTs with a total of 1831 patients that were subjected to qualitative and quantitative synthesis. In addition, 81 non-randomised studies were evaluated with regard to quality aspects and are summarised in Tables S1–S3 of the supplemental material.

### Trial characteristics and study population

As shown in Table 1, the six RCTs included in this meta-analysis were all published between 2002 and 2019, with three originating from Italy [23–25], two from Germany [9, 26] and one from Switzerland [27]. Across all RCTs, 1831 patients were randomised to open cut-down of the cephalic vein (*n* = 917) or closed cannulation of the subclavian vein (*n* = 914). The trial population was comparable in all of these RCTs: adult patients with oncological disease scheduled for TIVAP implantation for the implementation of chemotherapy (Table 1). The proportion of females varied from 32 [24] to 76% [23] in the open cut-down group and from 32 [24] to 79% [23] in the closed cannulation group. The patients’ mean age ranged from 52.1 years [23] to 64.7 years [25] in the open cut-down group and from 50.5 years [23] to 69.4 years [25] in the closed cannulation group. As expected, the open cut-down technique was performed exclusively by surgeons, while closed cannulation was performed by surgeons [9, 24, 25, 27] or radiologists [23, 26]. In the case of failure of open cut-down of the cephalic vein, most surgeons went on to perform puncture of the subclavian or jugular vein [23–25, 27]. Rescue techniques to avoid the risks of puncture were reported in two trials [9, 26]. In most cases, the subclavian vein was punctured using the landmark technique [24, 25, 27], while ultrasound guidance [23] and the roadmap technique [26] were the standard procedures in one study each. The multicentre trial by Hüttner et al. was designed pragmatically, allowing each centre to perform its standard procedures [9]. All procedures were generally performed in the operation room or angiographic suite with the patient under local anaesthesia. Antibiotic prophylaxis was administrated routinely in three trials [24, 25, 27], while the authors of other trials did not report routine antibiotic prophylaxis [23, 26].

**Table 1:** Characteristics of included randomised controlled trials comparing open cut-down of cephalic vein and closed cannulation of subclavian vein

Reference	Year	Country	Study type	Study period	Open cut-down of cephalic vein				Closed cannulation of subclavian vein					Primary endpoint
					*n*	Women	Mean age, years (SD)	Technique	*n*	Women	Mean age, years (SD)	Technique	Performer	
Hüttner [9]	2019	Germany	Multi-centre	2014–2016	583	296 (51%)	62.3 (11.9)	Standard venae sectio (first-line), rescue technique (second-line), puncture of subclavian vein (third line)	576	316 (55%)	61.1 (12.2)	Ultrasound, fluoroscopy or landmark technique (first-line), standard venae sectio (second-line), rescue technique (third line)	Surgeon	Pneumothorax/haematothorax
Knebel [26]	2011	Germany	Single centre	2008	51	31 (61%)	60.5 (11.5)	Standard venae sectio (first-line), rescue technique (second-line)	51	25 (49%)	57.4 (12.4)	Roadmap	Inter-ventional radiologist	Primary success
Biffi [23]	2009	Italy	Single centre	2003-2006	133	101 (76%)	52.1 (11.4)	Standard venae sectio (first-line), puncture (second-line)	136	108 (79%)	50.5 (12.0)	Ultrasound guidance	Radiologist	Peri- and postoperative complications
Nocito [27]	2009	Switzer-land	Single centre	2006-2008	76	51 (67%)	54.2 (9.6)	Standard venae sectio (first-line), puncture (second-line)	76	51 (67%)	56.0 (11.5)	Landmark	Surgeon	Primary success,
Rapisarda [25]	2006	Italy	Single centre	2000-2004	49	26 (53%)	64.7	Standard venae sectio (first-line), puncture (second-line)	50	26 (52%)	69.4	Landmark	Surgeon	Not stated
D’Angelo et al. [24]	2002	Italy	Single centre	1997-1998	25	8 (32%)	60	Standard venae sectio (first-line), puncture (second-line)	25	8 (32%)	63	Landmark	Surgeon	Not stated

### Methodological quality of included studies

**Fig. 2:**
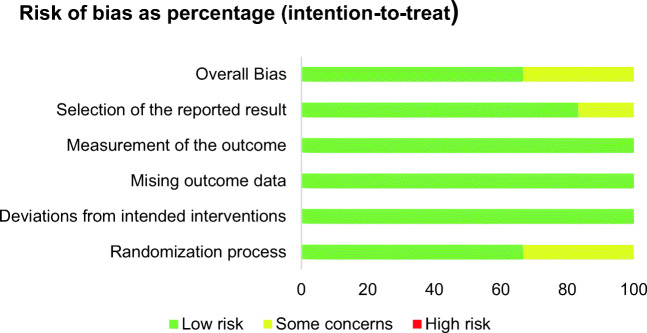
The risk of bias in the included randomised controlled trials for the primary endpoint pneumothorax (Cochrane risk of bias tool version 2)

The risk of bias according to RoB 2 is illustrated in Fig. 2, exemplarily for the primary endpoint, pneumothorax. In all six RCTs, bias was low with regard to ‘measurement of the outcome’, ‘missing outcome data’ and ‘deviations from intended interventions’. Some concerns arose in two trials [24, 25] regarding the ‘randomisation process’ and in one of these [25], there were also some concerns with respect to ‘the selection of the reported result’. Overall, bias was low in four RCTs [9, 23, 26, 27], while some concerns arose in two RCTs [24, 25].

### Quantitative analysis of the included RCTs

Quantitative analyses were performed for all outcomes with appropriate data from at least four of the included RCTs. For the outcomes early and late reintervention, early and late malfunction, TIVAP-associated infection, extravasation, nerve lesion, pinch-off phenomenon, TIVAP occlusion and hospital readmission, data from less than four RCTs were available and therefore had to be excluded from analysis. Summary statistics of meta-analyses of RCTs comparing the outcomes of open cut-down and closed cannulation are shown in Table 2 and described in detail in the following. Except for the outcomes ‘dislocation of the catheter/port chamber’ (*τ*^2^ = 0.386) and ‘failure of the primary technique’ (*τ*^2^ = 0.596), between-trial heterogeneity was assessed as zero for all outcomes.

**Table 2:** Summary statistics of meta-analyses of randomised controlled trials comparing outcomes of open cut-down and closed cannulation (puncture)

Outcome parameters (*N* = number of trials, *n* = number of patients)	Randomised controlled trials	
	OR [95% CI]	Between-study heterogeneity *τ*^*2*^
Pneumothorax (*N* = 6, *n* = 1818)	0.308 [0.122; 0.776]	0
Overall morbidity (*N* = 5, *n* = 1687)	0.903 [0.380; 2.147]	0
Mortality (*N* = 6, *n* = 1830)	1.183 [0.316; 4.431]	0
Dislocation of catheter or port chamber (*N* = 5, *n* = 1633)	2.519 [0.493; 12.876]	0.386
Thrombosis (*N* = 5, *n* = 1637)	0.771 [0.229; 2.599]	0
Postoperative bleeding/haematoma (*N* = 5, *n* = 1548)	0.853 [0.464; 1.569]	0
Failure of primary success (*N* = 6, *n* = 1831)	2.364 [1.051; 5.315]	0.596

Values are odds ratios (OR) with 95% confidence intervals (CI) and *τ*^2^ for the assessment of heterogeneity between the included studies, based on the continuity correction of 0.5 per cell

### Pneumothorax

**Fig. 3:**
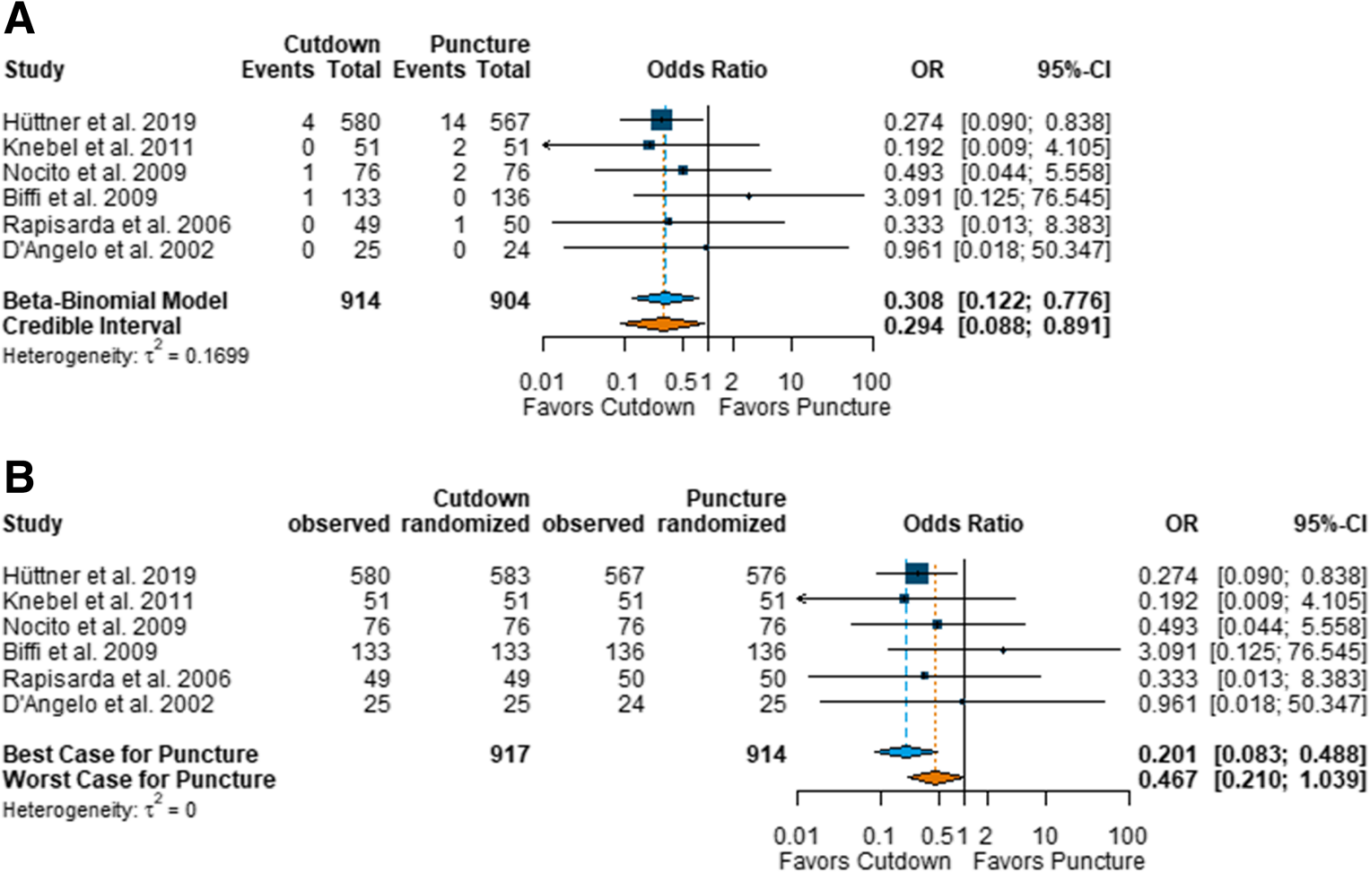
Forest plot of randomised controlled trials comparing pneumothorax in open cut-down and closed cannulation (puncture) techniques. **a** Blue diamond, primary analysis; orange diamond, credible interval. **b** Best-case/worst-case analysis

Pneumothorax was evaluated in all six RCTs, in a total of 1818 patients. Based on the ITT population, zero events were reported in three trials [24–26] for the open cut-down group and in two trials [23, 24] for the closed cannulation group. Meta-analysis showed significantly lower odds for the occurrence of a pneumothorax in the open cut-down group than in the closed cannulation group (OR 0.308, 95% CI 0.122 to 0.776) (Fig. 3a). Sensitivity analysis pooling OR and 95% credible interval confirmed the results from the beta-binomial model (OR 0.294, 95% CI 0.088 to 0.891) (Fig. 3a). The ‘worst-case’ scenario (in which all missing values in the open cut-down group were set to pneumothorax and all missing values in the closed cannulation group were set to no pneumothorax) showed a statistically non-significant effect in favour of the open cut-down technique (OR 0.467, 95% CI 0.210 to 1.039). The ‘best-case’ scenario (all missing values in the open cut-down group set to no pneumothorax and all missing values in the closed cannulation group set to pneumothorax) revealed a clear statistically significant effect in favour of the open cut-down technique group (OR 0.201, 95% CI 0.083 to 0.488) (Fig. 3b).

### Overall morbidity and mortality

All RCTs provided data on overall perioperative and postoperative morbidity. However, the RCT by Rapisarda et al. [25] was excluded from meta-analysis of morbidity due to a relevant discrepancy in the definition of this outcome parameter. In one trial, no morbidity events were observed in any of the study groups [24]. In quantitative analysis of 1687 patients, there was no statistically significant difference in overall morbidity between open cut-down and closed cannulation (OR 0.903, 95% CI 0.380 to 2.147). Mortality was reported in each RCT, with zero events in two trials [25, 27]. There was no statistically significant difference between the two procedures regarding mortality (OR 1.183, 95% CI 0.316 to 4.431).

### Dislocation of the catheter or port chamber

The occurrence of dislocation of the catheter or port chamber was reported in all RCTs, except the trial reported by Nocito and colleagues [27]. Zero events were frequently found for this outcome, in both the open cut-down group [24–26] and/or the closed cannulation group [23, 24]. Meta-analysis of 1633 patients showed an uncertain effect with a wide CI (OR 2.519, 95% CI 0.493 to 12.876).

### TIVAP-associated thrombosis

All but one RCT [27] assessed the rate of TIVAP-associated venous thrombosis. Zero events were reported in the RCT by D’Angelo and colleagues [24]. Quantitative analysis of 1637 patients revealed a statistically non-significant effect with a wide CI in favour of the open cut-down group (OR 0.771, 95% CI 0.229 to 2.599).

### Postoperative bleeding/haematoma

Postoperative bleeding and/or haematoma were reported in five RCTs including 1548 patients [9, 24–27]. One RCT reported zero events in both trial arms [24]. Pooling data from all five RCTs, there was no difference between open cut-down and closed cannulation regarding postoperative bleeding/haematoma (OR 0.853, 95% CI 0.464 to 1.569).

### Failure of the primary technique

**Fig. 4:**
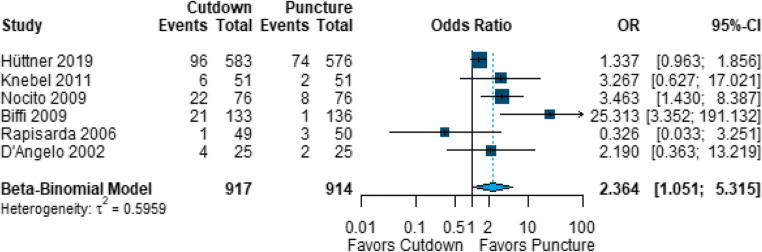
Forest plot of randomised controlled trials comparing rates of failure of the primary technique in open cut-down and closed cannulation (puncture) techniques

Each of the six RCTs reported the primary success rate. Figure 4 shows a forest plot comparing the rates of failure of the primary technique in the open cut-down and closed cannulation techniques. Failure of the primary technique occurred significantly more often with open cut-down than with closed cannulation procedures (OR 2.364, 95% CI 1.051 to 5.315).

### Publication bias

A funnel plot representative for the primary endpoint of pneumothorax in RCTs comparing open cut-down and closed cannulation is presented in Fig. S1. No clear publication bias was observed by visual inspection of the plot. Due to the relatively small number of RCTs (*n* < 10), no formal analysis for asymmetry was performed.

## Discussion

This systematic review and meta-analysis provide an update of critically appraised and quantitative data on the effectiveness and safety of open cut-down of the cephalic vein compared with closed cannulation of the subclavian vein for TIVAP implantation. Quantitative synthesis including six RCTs with a total of 1831 patients showed superiority of the primary open technique over closed cannulation with regard to the risk of pneumothorax, but lower primary success rates. There were no statistically significant differences between the two procedures regarding overall morbidity, mortality, dislocation of the catheter/port chamber, TIVAP-related thrombosis or postoperative bleeding/haematoma.

With approximately 14.1 million new cancer cases each year worldwide [28], the number of TIVAP implantations has increased significantly during the past 40 years. TIVAPs are widely accepted as an effective way of administering intravenous treatments, particularly chemotherapy in patients with oncological diseases. The use of TIVAPs has considerably improved patients’ comfort and safety [29]. Although TIVAP implantation is one of the most frequently performed procedures in oncology, no technique has yet been identified as reference standard with the best risk/benefit ratio [12, 23]. Open cut-down of the cephalic vein and closed cannulation of the subclavian vein are the two predominant procedures for TIVAP placement [7]. Both are safe and easy to perform; for anatomical reasons, however, only closed cannulation of the subclavian vein is associated with the risk of pneumothorax. There are further techniques like closed cannulation of internal jugular vein (IJV) which have to be excluded in this meta-analysis because not enough level 1 data is available at present [30–34]. Our updated meta-analysis supports the superiority of the primary open technique in the prevention of postoperative pneumothorax, as recently shown in the large multicentre PORTAS-3 trial [9]. This contradicts data from previous meta-analyses, published in 2014 and 2016 [7, 8], that showed similar frequencies of pneumothorax in patients undergoing open cut-down and closed cannulation for TIVAP implantation.

Our finding is of high clinical relevance, because pneumothorax is a serious complication that is associated with mortality and in many cases requires invasive treatment or admission to hospital [7, 9, 12, 35]. Furthermore, urgent oncological treatments, e.g. in haematological diseases, may be delayed due to this complication. The current meta-analysis has reached a sufficient sample size to detect a difference between the two procedures in the risk of pneumothorax; this difference was masked in previous meta-analyses by inappropriate sample sizes for the evaluation of infrequently occurring yet serious events [12]. Considering that data from six different trial populations are pooled in this meta-analysis, results can be generalised and are therewith transferable to daily practice.

The proponents of the closed cannulation technique often criticise the lower primary success rate of the open cut-down procedure, which explains the potential risk of pneumothorax in the event of conversion to puncture of the subclavian or jugular vein if open cut-down of the cephalic vein fails [7]. Indeed, the results from our meta-analysis confirm the superiority of closed cannulation over open cut-down in terms of primary success. Nevertheless, the primary success rate of open cut-down ranges between 71 and 98% in the six RCTs included in this meta-analysis [9, 23–27]. In two RCT [9, 26], a rescue technique by using the Seldinger method through the cephalic vein during open surgery was able to increase the primary success rate of the open cut-down up to over 90%. The primary success rate of the closed cannulation technique ranges between 87 and 99% in the same studies [9, 23–27]. Therefore, a rather low increase in primary success rate entails a three times higher risk of pneumothorax. In this context, the reliable avoidance of pneumothorax by open cut-down justifies its use as reference standard for TIVAP implantation [9]. As shown in the PORTAS-1 trial, the use of the Seldinger method as a rescue strategy during open surgery can further avoid puncture of the subclavian vein due to failure of the open cut-down technique [36]. Additionally, patients with underlying oncological disease may benefit not only from reduced numbers of complications but also from improved survival and recurrence rates when chemotherapy cycles are not delayed or interrupted because of serious problems after TIVAP implantation by other means. Considering the increased health-care costs associated with peri-interventional and post-interventional complications [37], and the large number of TIVAP implantations performed worldwide, the prevention of severe complications is also of socio-economic relevance. More than 400,000 TIVAPs are sold annually in the USA—illustrating the number of patients treated each year. Based on the event rates reported in the six RCTs included in this meta-analysis, the number needed to treat is 72 for the primary outcome. This means that 72 patients must undergo open cut-down of the cephalic vein to prevent a pneumothorax in one patient. Therefore, more than 5,555 pneumothorax events could be prevented in the USA per year [38].

One of the strengths of this meta-analysis is its large sample size: six RCTs with a total number of 1831 patients. In contrast to the meta-analysis by Orci et al. including the same number of RCTs with less than the half of patients (*n* = 772) and a recent Cochrane review [8], we excluded the study by Boldo et al. [39], in which not only the subclavian vein but also the internal jugular vein was used for closed cannulation, but the specific vessel was not reported regarding event rates, so the trial population was too heterogeneous to meet our inclusion criteria. In contrast to previous meta-analyses, we have consequently pooled ITT data from primary studies, reflecting the practical clinical scenario. To account for the low rates of complications with zero events in numerous RCTs, the beta-binomial model was applied, as continuity correction should be avoided [16]. These results were confirmed by fitting a Bayesian random-effects meta-analysis to the data of the primary endpoint [16, 17]. Even the best-case/worst-case analysis strongly supports the results of the primary analysis.

The main weakness of this meta-analysis is its deviation from the study protocol published in 2015 [12]. The originally conceived purpose of this study was to summarise the available evidence on all perioperative complications of open cut-down of the cephalic vein compared with closed cannulation of the subclavian vein. For this purpose, naïve pooling was planned for each outcome using data from both non-comparative and comparative studies. Because the number of patients included in RCTs is now considerably higher [9], we modified the statistical analysis plan to make it more stringent, including only RCTs in quantitative analysis. We are convinced that the inclusion of non-randomised studies in this situation would incur considerable risk of bias and would not add any additional reliable evidence. Consequently, we included non-randomised studies only in qualitative analysis and present an overview of these studies in the form of supplemental tables. Despite the greatly increased risk of bias, even the results of non-randomised studies show superiority of the open cut-down technique regarding the risk of pneumothorax compared with closed cannulation [40].

A further limitation of this meta-analysis is the heterogeneity in the closed cannulation group regarding guidance. Three trials used blind puncture, also called landmark technique, for closed cannulation only. Whereas one trial used ultrasound guidance, one used contrast agent, called roadmap technique, and one trial used a mixture of ultrasound and landmark technique (Table 1). The pneumothorax frequencies were 2% for blind puncture, 0% for ultrasound guidance, 3,9% for roadmap technique and 2,5% for the mixed technique group. The trial by Biffi et al. using ultrasound guidance on every patient in the closed cannulation group showed the lowest pneumothorax frequency [23]. Therefore, a significant lower pneumothorax frequency could be postulated if ultrasound would be used consequently as guidance for closed cannulation. However, there is a Cochrane meta-analysis from 2015 comparing ultrasound guidance with anatomical landmark technique in subclavian and femoral vein catheterisation which reported reduced inadvertent arterial cannulations and hematoma formation but failed to show improved success or reduced overall complications rates including pneumothorax or haemothorax [41]. Another meta-analysis from 2015 stated that even sonographic guidance is not able to reduce pneumothorax risk to zero [42]. Therefore, the significance and impact of ultrasound guidance on pneumothorax frequency remain unclear. Further research would be necessary. Given the inherent need for a surgical incision to implant the port chamber placement of the catheter under direct vision into the cephalic vein via this incision seems most natural and if successful, there is no risk at all for pneumo- and/or haemothorax.

In conclusion, this meta-analysis provides updated level I evidence that primary open cut-down of the cephalic vein is superior to closed cannulation of the subclavian vein regarding the risk of pneumothorax. Therefore, we would recommend for TIVAP implantation the open cut-down technique as first-line approach and ultrasound guided–closed cannulation as second-line approach if open cut-down fails.

## Supplementary information

ESM 1 (DOCX 233 kb)

ESM 2 (DOC 64 kb)

Fig. S1 Funnel plot for the endpoint pneumothorax (PNG 4948 kb)

High resolution (TIFF 1142 kb)

## Abbreviations

CI: confidence interval
ITT: intention-to-treat
OR: odds ratio
PRISMA: Preferred Reporting Items for Systematic Reviews and Meta-Analyses
RCT: randomised controlled trial
ROB 2: Cochrane risk of bias tool version 2
TIVAP: totally implantable venous access port
