# Supplementary material for: Meta-analysis of primary open versus closed cannulation strategy for totally implantable venous access port implantation
Source: Langenbecks Arch Surg. 2021 Jan 9;406(3):587–96. doi: 10.1007/s00423-020-02057-w (PMC8106576; doi:10.1007/s00423-020-02057-w)
Supplement: Supplementary file 1 — (DOCX 233 kb) [file 423_2020_2057_MOESM1_ESM.docx]

**Non-randomised studies investigating open cut-down of cephalic vein and/or closed cannulation of subclavian vein**

***Selection of non-randomised studies***

Non-randomised studies with at least one intervention group investigating peri- and postoperative outcomes of patients undergoing TIVAP implantation by either open cut-down of the cephalic vein or closed cannulation of the subclavian vein were eligible. Case reports, studies investigating TIVAP implantation in children (patients <15 years of age), and studies focusing on patients with rare diseases such as cystic fibrosis, sickle cell anemia, immunodeficiency syndrome and other non-oncological diseases with impaired immune or coagulation system were excluded.

***Assessment of methodological quality of included non-randomised studies***

For non-randomised studies, the methodological quality of the studies included was assessed by means of a critical appraisal tool comprising questions on the quality of adverse effects assessment and reporting as described by Loke et al. ([1](#_ENREF_1)). Risk of bias in non-randomised studies was classified as ‘low’, ‘moderate’ or ‘high’.

***Characteristics of non-randomised studies included***

Eighty-one non-randomised studies (i.e. non-randomised with regard to open cut-down of the cephalic vein and closed cannulation of the subclavian vein) were included and evaluated with regard to quality aspects and summarised via tabulation in Tables S1-S3. As shown in Table S1, 12 CCTs compared open cut-down of the cephalic vein with closed cannulation of the subclavian vein. In 69 studies, one of the two procedures was reported: open cut-down of the cephalic vein in 16 studies (Table S2) and closed cannulation of the subclavian vein in 53 studies (Table S3). Included non-randomised studies were published between 1985 and 2019 originating from Europe, North America, South America, Asia and Australia. Sample sizes ranged from 30 ([2](#_ENREF_2)) to 8,654 ([3](#_ENREF_3)) patients in individual studies. All but two studies ([4](#_ENREF_4), [5](#_ENREF_5)) were monocentric. Sixty out of 81 studies (74%) were designed retrospectively, whereas in 21 studies (26%), data were assessed prospectively. Risk of bias in non-randomised studies was ‘moderate’ in 63 out of 81 studies (78%), whereas ‘low’ and ‘high risk’ of bias was present in 15 (19%) ([6-19](#_ENREF_6)) and 3 studies (4%) ([2](#_ENREF_2), [20](#_ENREF_20), [21](#_ENREF_21)), respectively (Tables S1-S3).

**Table S1** Characteristics of included non-randomised studies comparing open cut-down of cephalic vein and closed cannulation of subclavian vein.

| **Reference** | **Country** | **Study type** | **Study period** | **Open cut-down of cephalic vein^£^** | | | | **Closed cannulation of subclavian vein** | | | | | **Risk of bias** |
| --- | --- | --- | --- | --- | --- | --- | --- | --- | --- | --- | --- | --- | --- |
|  |  |  |  | **n** | **Women** | **Mean age (years)** | **Technique** | **n** | **Women** | **Mean age**  **(years)** | **Technique** | **Performer** |  |
| **Alsfasser (**[**22**](#_ENREF_22)**)**  **2014** | Germany | CCT  Retro-spective  Single-center | 1998-2008 | 101 | ns | ns | Standard venae sectio | 1,322 | ns | ns | Landmark | Surgeon | Moderate |
| **Aspalter 2014 (**[**23**](#_ENREF_23)**)** | Austria | CCT  Retro-spective  Single-center | 2009-2011 | 307 | ns | ns | Standard venae sectio (1^st^ line)  Puncture (2^nd^ line) | 300 | ns | ns | Landmark | Surgeon | Moderate |
| **Chang 2006 (**[**24**](#_ENREF_24)**)** | Taiwan | CCT  Retro-spective  Single-center | 1998-2003 | 533 | 205 (38%) | 60 | Standard venae sectio | 598 | 286 (48%) | 64 | Landmark | Surgeon | Moderate |
| **El Hammoumi 2014 (**[**25**](#_ENREF_25)**)** | Morocco | CCT  Retro-spective  Single-center | 2002-2013 | 452 | ns | ns | Standard venae sectio | 1,008 | ns | ns | Landmark  (ultrasound guidance in 52 patients) | Surgeon | Moderate |
| **Granziera 2014 (**[**18**](#_ENREF_18)**)** | Italy | CCT  Retro-spective  Single-center | 2006-2008 | 102 | 50 (49%) | ns | Standard venae sectio | 48 | 33 (69%) | ns | Landmark | Other | Low |
| **Haaf 2014 (**[**19**](#_ENREF_19)**)** | Germany | CCT  Pro-spective  Single-center | One year | 53 | 53 (100%) | ns | Standard venae sectio | 57 | 55 (96%) | ns | Landmark | Other | Low |
| **Jablon 2006**  **(**[**26**](#_ENREF_26)**)** | USA | CCT  Retro-spective  Single-center | 1998-2001 | 148 | ns | ns | Standard venae section (1^st^ line)  Rescue technique (2^nd^ line) | 163 | ns | ns | Landmark | Surgeon | Moderate |
| **Matiotti-Neto 2017 (**[**27**](#_ENREF_27)**)** | USA | CCT  Retro-spective  Single-center | 2010-2012 | 195 | 118 (61%) | ns | Standard venae sectio (1^st^ line)  Rescue technique (2^nd^ line) | 247 | 145 (59%) | ns | Landmark | Surgeon | Moderate |
| **Ragusa 2000 (**[**28**](#_ENREF_28)**)** | Italy | CCT  Retro-spective  Single-center | 1994-1998 | 147 | ns | ns | Standard venae sectio (1^st^ line)  Rescue technique (2^nd^ line) | 48 | ns | ns | Landmark | Surgeon | Moderate |
| **Rhu**  **2019 (**[**29**](#_ENREF_29)**)** | Korea | CCT  Retro-spective  Single-center | 2012-2014 | 119 | 68 (57%) | 58 | Standard venae sectio | 230 | 153 (67%) | 56 | Landmark | Surgeon | Moderate |
| **Otsubo 2016 (**[**30**](#_ENREF_30)**)** | Japan | CCT  Retro-spective  Single-center | 2011-2013 | 149 | 77 (52%) | 70 | Standard venae sectio +/- pre-operative ultra-sono-graphy (1^st^ line)  Puncture (2^nd^ line) | 122 | 68 (56%) | 67 | Landmark | Surgeon | Moderate |
| **Vetter 2013 (**[**31**](#_ENREF_31)**)** | Germany | CCT  Retro-spective  Single-center | 2005-2006 | 51 | 22 (43%) | 51) | Standard venae sectio | 32 | 11 (34%) | 53 | Landmark | Surgeon | Moderate |

CS: case series, CCT: controlled clinical trials, RCT: randomized controlled trial; n: sample size; *as regards the average number of procedures per month; ns: not stated; mean age in years; ^£^all procedures performed by surgeons

**Table S2** Characteristics of included non-randomised studies investigating open cut-down of cephalic vein.

| **Reference** | **Country** | **Study type** | **Study period** | **n** | **Women** | **Mean age**  **(years)** | **Technique** | **Performer** | **Risk of bias** |
| --- | --- | --- | --- | --- | --- | --- | --- | --- | --- |
| **Ayadi 2011 (**[**32**](#_ENREF_32)**)** | Tunis | CS  Pro-  spective  Single-center | 2009-1010 | 58 | 32 (55%) | 52 | Standard venae sectio (1^st^ line)  Puncture (2^nd^ line) | Surgeon | Moderate |
| **Conessa 2002 (**[**33**](#_ENREF_33)**)** | France | CS  Retro-spective  Single-center | 1997-2001 | 105 | 18 (17%) | 59 | Standard venae sectio (1^st^ line)  Puncture (2^nd^ line) | Surgeon | Moderate |
| **Di Carlo 2001 (**[**34**](#_ENREF_34)**)** | Italy | CS  Retro-spective  Single-center | 1995-1999 | 344 | 141 (41%) | ns | Standard venae sectio | Surgeon | Moderate |
| **Goossens 2013 (**[**16**](#_ENREF_16)**)** | Belgium | RCT  Pro-  spective  Single-center | 2009-2010 | 802 | 524 (65%) | na | Standard venae sectio (1^st^ line)  Puncture (2^nd^ line) | Surgeon | Low |
| **Hashimoto 2019 (**[**35**](#_ENREF_35)**)** | Japan | CCT  Retro-spective  Single-center | 2015-2017 | 212 | 111 (52%) | 67 | Standard venae sectio (1^st^ line)  Rescue technique (2^nd^ line)  Puncture (3^rd^ line) | Surgeons | Moderate |
| **Hoareau-Gruchet 2009 (**[**36**](#_ENREF_36)**)** | France | CS  Pro-  spective  Single-center | 2005-2008 | 122 | 19 (16%) | 59 | Standard venae sectio | Surgeon | Moderate |
| **Horng 2007 (**[**37**](#_ENREF_37)**)** | Taiwan | CCT  Retro-spective  Single-center | 2004-2005 | 158 | 158 (100%) | na | Standard venae sectio | Surgeon | Moderate |
| **Iorio 2018 (**[**38**](#_ENREF_38)**)** | Italy | CCT  Pro-  spective  Single-center | 2016 | 109 | 45 (41%) | 61 | Standard venae sectio | Surgeon | Moderate |
| **Knebel 2009 (**[**20**](#_ENREF_20)**)** | Germany | RCT  Pro-  spective  Single-center | 2006-2007 | 164 | 74 (45%) | na | Standard venae sectio vs. rescue technique | Surgeon | High |
| **Marcy 2005 (**[**39**](#_ENREF_39)**)** | France | CCT  Retro-spective  Single-center | 1998-2001 | 100 | 100 (100%) | 55 | Standard venae sectio | Surgeon | Moderate |
| **Povoski 2000 (**[**40**](#_ENREF_40)**)** | USA | CS  Pro-  spective  Single-center | 1998-1999 | 56 | ns | ns | Standard venae sectio | Surgeon | Moderate |
| **Repelaer van driel 1988 (**[**41**](#_ENREF_41)**)** | Netherlands | CS  Retro-spective  Single-center | 1984-1986 | 45 | ns | ns | Standard venae sectio | Surgeon | Moderate |
| **Sarzo 2004 (**[**42**](#_ENREF_42)**)** | Italy | CS  Retro-spective  Single-center | 2001-2002 | 106 | 44 (42%) | 59 | Standard venae sectio | Surgeon | Moderate |
| **Schumacher 2007 (**[**43**](#_ENREF_43)**)** | Germany | CS  Retro-spective  Single-center | 1995-2006 | 3,498 | 1889 (54%) | 57 | Standard venae sectio (1^st^ line)  Puncture (2^nd^ line) | Surgeon | Moderate |
| **Seiler 2006 (**[**17**](#_ENREF_17)**)** | Germany | CS  Retro-spective  Single-center | 2001-2003 | 400 | 270 (68%) | 56 | Standard venae sectio (1^st^ line)  Puncture (2^nd^ line) | Surgeon | Low |
| **Wenke 1990 (**[**44**](#_ENREF_44)**)** | Germany | CS  Retro-spective  Single-center | 1984-1989 | 82 | 58 (71%) | 52 | Standard venaesectio (1^st^ line)  Puncture (2^nd^ line) | Surgeon | Moderate |

CS: case series, CCT: controlled clinical trials, RCT: randomized controlled trial; n: sample size; ns: not stated; na: not applicable; mean age in year

**Table S3** Characteristics of included non-randomised studies investigating closed cannulation of subclavian vein.

| **Reference** | **Country** | **Study type** | **Study period** | **n** | **Women** | **Mean age**  **(years)** | **Technique** | **Performer** | **Risk of bias** |
| --- | --- | --- | --- | --- | --- | --- | --- | --- | --- |
| **Adamus 2002 (**[**45**](#_ENREF_45)**)** | Germany | CS  Retro-spective  Single-center | 1999-2001 | 41 | 21 (51%) | 61 | Ultrasound guidance | Radiologist | Moderate |
| **Akahane 2011 (**[**6**](#_ENREF_6)**)** | Japan | CCT  Retro-spective  Single-center | 2006-2009 | 47 | 7 (15%) | ns | Ultrasound guidance | Radiologist | Low |
| **Aldrighetti 2000 (**[**7**](#_ENREF_7)**)** | Italy | CS  Pro-spective  Single-center | 1991-1999 | 967 | 443 (46%) | 56 | Landmark | Surgeon | Low |
| **Apsner 2001**  **(**[**46**](#_ENREF_46)**)** | Austria | CS  Pro-spective  Single-center | Two years | 101 | 57 (56%) | 55 | Landmark | Other | Moderate |
| **Araujo 2008 (**[**47**](#_ENREF_47)**)** | Portugal | CCT  Pro-spective  Single-center | 2003-2006 | 617 | 384 (62%) | ns | Landmark | Surgeon | Moderate |
| **Aziret**  **2015 (**[**48**](#_ENREF_48)**)** | Turkey | CCT  Pro-spective  Single-center | 2012-2014 | 122 | 55 (45%) | 57.9 | Landmark | Surgeon | Moderate |
| **Bademler 2019 (**[**49**](#_ENREF_49)**)** | Turkey | CS  Retro-spective  Single-center | 2010-2017 | 2,628 | 1,247 | ns | Ultrasound guidance/ landmark/ | Surgeon | Moderate |
| **Ballarini 1999 (**[**50**](#_ENREF_50)**)** | Italy | CS  Retro-spective  Single-center | 1992-1997 | 99 | ns | ns | Landmark | Surgeon | Moderate |
| **Barbetakis 2011 (**[**8**](#_ENREF_8)**)** | Greece | CS  Retro-spective  Single-center | 2001-2010 | 700 | 498 (71%) | 55 | Landmark | Surgeon | Low |
| **Barrios 1992 (**[**51**](#_ENREF_51)**)** | USA | CS  Retro-spective  Single-center | 1985-1988 | 218 | ns | ns | Landmark | Surgeon | Moderate |
| **Biffi 1998 (**[**52**](#_ENREF_52)**)** | Italy | CS  Pro-spective  Single-center | 1994-1997 | 328 | 200 (61%) | ns | Landmark | Surgeon | Moderate |
| **Brooks 2005 (**[**53**](#_ENREF_53)**)** | Australia | CS  Retro-spective  Single-center | 1998-2002 | 55 | ns | ns | Ultrasound guidance | Surgeon | Moderate |
| **Campisi 1997 (**[**54**](#_ENREF_54)**)** | Italy | CS  Retro-spective  Single-center | Two years | 201 | 132 (66%) | ns | Landmark | Surgeon | Moderate |
| **Capaccioli 1998 (**[**55**](#_ENREF_55)**)** | Italy | CS  Retro-spective  Single-center | 1993-1997 | 87 | 61 (70%) | 55 | Landmark | Radiologist | Moderate |
| **Chang 2012 (**[**56**](#_ENREF_56)**)** | Germany | CS  Retro-spective  Single-center | 2006-2010 | 1,532 | 1127 (74%) | 56 | Ultrasound guidance | Radiologist | Moderate |
| **de Gregorio 1996. (**[**57**](#_ENREF_57)**)** | Spain | CS  Retro-spective  Single-center | 1989-1993 | 288 | 128 (44%) | 55 | Landmark | Radiologist | Moderate |
| **Florio 2008 (**[**4**](#_ENREF_4)**)** | Italy | CS  Retro-spective  Multi-center | 2004-2007 | 108 | ns | ns | Landmark | Radiologist | Moderate |
| **Garajová 2012 (**[**58**](#_ENREF_58)**)** | Italy | CS  Retro-spective  Single-center | 1997-2011 | 252 | 108 (43%) | ns | Landmark | Surgeon | Moderate |
| **Güner 2013 (**[**59**](#_ENREF_59)**)** | Turkey | CS  Retro-spective  Single-center | 2006-2009 | 238 | 109 (46%) | 58 | Landmark | Surgeon | Moderate |
| **Heibl 2010 (**[**60**](#_ENREF_60)**)** | Austria | CS  Pro-spective  Single-center | 2004 | 140 | 61 (44%) | ns | Landmark | Surgeon | Moderate |
| **Herrmann 1999 (**[**61**](#_ENREF_61)**)** | Germany | CS  Retro-spective  Single-center | 1997-1999 | 53 | 45 (85%) | 54 | Landmark | Radiologist | Moderate |
| **Karanlik 2009 (**[**62**](#_ENREF_62)**)** | Turkey | CCT  Pro-spective  Single-center | 2007-2008 | 451 | 205 (45%) | na | Landmark | Surgeon | Moderate |
| **Karanlik 2011 (**[**9**](#_ENREF_9)**)** | Turkey | RCT  Pro-spective  Single-center | 2008-2009 | 404 | 207 (51%) | na | Landmark | Surgeon | Low |
| **Keum 2013 (**[**63**](#_ENREF_63)**)** | Korea | CS  Retro-spective  Single-center | 2009-2010 | 242 | 160 (66%) | 56 | Landmark | Surgeon | Moderate |
| **Kluge 1998 (**[**64**](#_ENREF_64)**)** | Germany | CS  Retro-spective  Single-center | 1994-1997 | 120 | 68 (57%) | 55 | Landmark | Radiologist | Moderate |
| **Koonings 1994 (**[**65**](#_ENREF_65)**)** | USA | CS  Retro-spective  Single-center | 1984-1992 | 100 | 100 (100%) | 59 | Landmark | Surgeon | Moderate |
| **Ku 2009 (**[**10**](#_ENREF_10)**)** | Taiwan | CS  Retro-spective  Single-center | 2004-2007 | 1,025 | 465 (45%) | 57 | Landmark | Surgeon | Low |
| **Leclerc 1987 (**[**66**](#_ENREF_66)**)** | Canada | CS  Retro-spective  Single-center | 1984-1986 | 47 | 25 (52%) | 49 | Landmark | Surgeon | Moderate |
| **Leinung 2002 (**[**67**](#_ENREF_67)**)** | Germany | CS  Retro-spective  Single-center | 1999-2000 | 311 | ns | ns | Landmark | Surgeon | Moderate |
| **Lokich 1985**  **(**[**68**](#_ENREF_68)**)** | USA | CS  Retro-spective  Single-center | 1982-1983 | 92 | ns | ns | Landmark | Surgeon | Moderate |
| **Lorch 2001 (**[**69**](#_ENREF_69)**)** | Germany | CS  Retro-spective  Single-center | 1997-1998 | 123 | 55 (45%) | 57 | Roadmap | Radiologist | Moderate |
| **Matsushima (**[**70**](#_ENREF_70)**)**  **2017** | Japan | CS  Retro-spective  Singel-center | 2010-2016 | 82 | ns | ns | Landmark | Surgeon | Moderate |
| **Miao 2014 (**[**71**](#_ENREF_71)**)** | China | RCT  Pro-spective  Single-center | 2009-2013 | 107 | 88 (82%) | 59 | Landmark | Surgeon | Moderate |
| **Nagasawa 2014 (**[**11**](#_ENREF_11)**)** | Japan | CCT  Retro-spective  Single-center | 2007-2012 | 100 | 37 (37%) | ns | Landmark | Surgeon | Low |
| **Nelson 1994 (**[**72**](#_ENREF_72)**)** | USA | CS  Retrospective  Single-center | 1989-1991 | 67 | 67 (100%) | 50 | Landmark | Surgeon | Moderate |
| **Orsi 2000 (**[**73**](#_ENREF_73)**)** | Italy | CCT  Retro-spective  Single-center | 1996-1999 | 427 | 290 (68%) | 53 | Landmark vs. ultrasound guidance | Surgeon | Moderate |
| **Ozyuvaci 2006 (**[**74**](#_ENREF_74)**)** | Turkey | CS  Retro-spective  Single-center | 2001-2006 | 368 | 270 (73%) | ns | Landmark | Other | Moderate |
| **Paprottka 2019 (**[**3**](#_ENREF_3)**)** | Germany | CS  Retro-spective  Single-center | 1998-2014 | 8,654 | 4,686 (54%) | ns | Landmark | Radiologist | Moderate |
| **Plumhans 2011 (**[**75**](#_ENREF_75)**)** | Germany | CCT  Pro-spective  Single-center | 2007-2008 | 94 | ns | ns | Roadmap | Radiologist | Moderate |
| **Poorter 1996 (**[**76**](#_ENREF_76)**)** | Netherlands | CS  Retro-spective  Single-center | 1989-1995 | 149 | 64 (43%) | ns | Landmark | Surgeon | Moderate |
| **Sakamoto 2010 (**[**12**](#_ENREF_12)**)** | Japan | CS  Retro-spective  Single-center | 2006-2007 | 486 | 221 (45%) | 54 | Ultrasound guidance | Radiologist | Low |
| **Shen-Gunther 2003 (**[**77**](#_ENREF_77)**)** | USA | CS  Retro-spective  Single-center | 1996 | 49 | 49 (100%) | ns | Landmark | Surgeon | Moderate |
| **Shetty 1997 (**[**78**](#_ENREF_78)**)** | USA | CS  Retro-spective  Single-center | 1993-1996 | 346 | 220 (64%) | 56 | Roadmap | Radiologist | Moderate |
| **Shiono 2014 (**[**79**](#_ENREF_79)**)** | Japan | CCT  Retro-spective  Single-center | 2006-2011 | 342 | 130 (38%) | ns | Ultrasound guidance | Other | Moderate |
| **Simpson 1997 (**[**80**](#_ENREF_80)**)** | USA | CS  Retro-spective  Single-center | 1993-1995 | 157 | 63 (40%) | 57 | Roadmap  (95% of patients) | Radiologist | Moderate |
| **Sutor 2012 (**[**21**](#_ENREF_21)**)** | Germany | CS  Retro-spective  Single-center | 2009-2010 | 50 | 9 (18%) | ns | Ultrasound guidance | Surgeon | High |
| **Süslü 2012 (**[**81**](#_ENREF_81)**)** | Turkey | CS  Retro-spective  Single-center | 2006-2008 | 82 | 52 (63%) | ns | Landmark | Other | Moderate |
| **Tagliari 2015 (**[**82**](#_ENREF_82)**)** | Brazil | RCT  Pro-spective  Single-center | 2014-2015 | 35 | 17 (49%) | 55 | Landmark | Surgeon | Low |
| **Tsai 2012 (**[**13**](#_ENREF_13)**)** | Taiwan | CCT  Retro-spective  Single-center | 2004-2008 | 1,848 | 821 (44%) | na | Landmark | Surgeon | Low |
| **Vandoni 2009 (**[**14**](#_ENREF_14)**)** | Switzerland | RCT  Pro-spective  Single-center | 1998-2001 | 228 | 132 (58%) | 58 | Landmark | Surgeon | Low |
| **Vardy 2004 (**[**5**](#_ENREF_5)**)** | Australia | CS  Pro-spective  Multi-center | 1998-2001 | 110 | 64 (58%) | ns | Roadmap | Radiologist | Moderate |
| **Welling 1986 (**[**2**](#_ENREF_2)**)** | USA | CS  Retro-spective  Single-center | 1983-1985 | 30 | 25 (83%) | ns | Landmark | Surgeon | High |
| **Zähringer 2006 (**[**15**](#_ENREF_15)**)** | Germany | CS  Retro-spective  Single-center | 2003-2004 | 271 | 186 (69%) | 53 | Ultrasound guidance | Radiologist | Low |

CS: case series, CCT: controlled clinical trials, RCT: randomized controlled trial; n: sample size; *as regards the average number of procedures per month; ns: not stated; na: not applicable; mean age in years

**References**

1. Loke YK, Price D, Herxheimer A. Systematic reviews of adverse effects: framework for a structured approach. BMC Med Res Methodol. 2007;7:32.

2. Welling RE, Hall JM, Meyer RL, Arbaugh JJ. Implantable venous access devices: an alternative method of extended cancer care. Journal of surgical oncology. 1986;33(2):73-5.

3. Paprottka KJ, Voelklein J, Waggershauser T, Reiser MF, Paprottka PM. Retrospective outcome analysis of rates and types of complications after 8654 minimally invasive radiological port implantations via the subclavian vein without ultrasound guidance. La Radiologia medica. 2019;124(9):926-33.

4. Florio G, Del Papa M, Mari A, Carni D. Totally implantable central venous access devices in adult oncological patients. Chirurgia italiana. 2008;60(5):697-702.

5. Vardy J, Engelhardt K, Cox K, Jacquet J, McDade A, Boyer M, et al. Long-term outcome of radiological-guided insertion of implanted central venous access port devices (CVAPD) for the delivery of chemotherapy in cancer patients: institutional experience and review of the literature. British journal of cancer. 2004;91(6):1045-9.

6. Akahane A, Sone M, Ehara S, Kato K, Tanaka R, Nakasato T. Subclavian vein versus arm vein for totally implantable central venous port for patients with head and neck cancer: a retrospective comparative analysis. Cardiovascular and interventional radiology. 2011;34(6):1222-9.

7. Aldrighetti L, Paganelli M, Arru M, Caterini R, Ronzoni M, Ferla G. Complications of blind placement technique in 980 subcutaneous infusion ports. The journal of vascular access. 2000;1(1):28-32.

8. Barbetakis N, Asteriou C, Kleontas A, Tsilikas C. Totally implantable central venous access ports. Analysis of 700 cases. Journal of surgical oncology. 2011;104(6):654-6.

9. Karanlik H, Kurul S, Saip P, Unal ES, Sen F, Disci R, et al. The role of antibiotic prophylaxis in totally implantable venous access device placement: results of a single-center prospective randomized trial. American journal of surgery. 2011;202(1):10-5.

10. Ku YH, Kuo PH, Tsai YF, Huang WT, Lin MH, Tsao CJ. Port-A-Cath implantation using percutaneous puncture without guidance. Annals of surgical oncology. 2009;16(3):729-34.

11. Nagasawa Y, Shimizu T, Sonoda H, Mekata E, Wakabayashi M, Ohta H, et al. A comparison of outcomes and complications of totally implantable access port through the internal jugular vein versus the subclavian vein. International surgery. 2014;99(2):182-8.

12. Sakamoto N, Arai Y, Takeuchi Y, Takahashi M, Tsurusaki M, Sugimuta K. Ultrasound-Guided Radiological Placement of Central Venous Port via the Subclavian Vein: A Retrospective Analysis of 500 Cases at a Single Institute. Cardiovascular and interventional radiology. 2010;33(5):989-94.

13. Tsai YF, Ku YH, Chen SW, Huang WT, Lu CC, Tsao CJ. Right- and left-subclavian vein port-a-cath systems: comparison of complications. European surgical research Europaische chirurgische Forschung Recherches chirurgicales europeennes. 2012;49(2):66-72.

14. Vandoni RE, Guerra A, Sanna P, Bogen M, Cavalli F, Gertsch P. Randomised comparison of complications from three different permanent central venous access systems. Swiss medical weekly. 2009;139(21-22):313-6.

15. Zahringer M, Hilgers J, Kruger K, Strohe D, Bangard C, Neumann L, et al. [Ultrasound guided implantation of chest port systems via the lateral subclavian vein]. RoFo : Fortschritte auf dem Gebiete der Rontgenstrahlen und der Nuklearmedizin. 2006;178(3):324-9.

16. Goossens GA, Jerome M, Janssens C, Peetermans WE, Fieuws S, Moons P, et al. Comparing normal saline versus diluted heparin to lock non-valved totally implantable venous access devices in cancer patients: a randomised, non-inferiority, open trial. Annals of oncology : official journal of the European Society for Medical Oncology / ESMO. 2013;24(7):1892-9.

17. Seiler CM, Frohlich BE, Dorsam UJ, Kienle P, Buchler MW, Knaebel HP. Surgical technique for totally implantable access ports (TIAP) needs improvement: a multivariate analysis of 400 patients. Journal of surgical oncology. 2006;93(1):24-9.

18. Granziera E, Scarpa M, Ciccarese A, Filip B, Cagol M, Manfredi V, et al. Totally implantable venous access devices: retrospective analysis of different insertion techniques and predictors of complications in 796 devices implanted in a single institution. BMC surgery. 2014;14:27.

19. Haaf O, Termath-Bethge B, Sauerwald A, Wittmann M, Hohn A, Winkels J, et al. [Process-related observations on the placement of port-catheter systems by anaesthetists]. Anästhesiologie & Intensivmedizin. 2014;55(12):634.

20. Knebel P, Fischer L, Huesing J, Hennes R, Buchler MW, Seiler CM. Randomized clinical trial of a modified Seldinger technique for open central venous cannulation for implantable access devices. The British journal of surgery. 2009;96(2):159-65.

21. Sutor T, Schmidt H, Stasche N. [First experiences with ultrasound guided subclavian vein port placement in ENT]. Laryngo- rhino- otologie. 2012;91(1):28-31.

22. Alsfasser G, Neumann A, Klar E, Eisold S. [Venous Access Port Implantation is an Ideal Teaching Operation - An Analysis of 1423 Cases.]. Zentralblatt fur Chirurgie. 2014.

23. Aspalter M, Lechner M, Linni K, Hitzl W, Holzenbein T, Ofner D, et al. Morbidity after insertion of totally implantable venous access ports in oncological patients: results of a retrospective clinical study. The American surgeon. 2014;80(2):204-7.

24. Chang HM, Hsieh CB, Hsieh HF, Chen TW, Chen CJ, Chan DC, et al. An alternative technique for totally implantable central venous access devices. A retrospective study of 1311 cases. European journal of surgical oncology : the journal of the European Society of Surgical Oncology and the British Association of Surgical Oncology. 2006;32(1):90-3.

25. El Hammoumi M, El Ouazni M, Arsalane A, El Oueriachi F, Mansouri H, Kabiri el H. Incidents and complications of permanent venous central access systems: a series of 1,460 cases. The Korean journal of thoracic and cardiovascular surgery. 2014;47(2):117-23.

26. Jablon LK, Ugolini KR, Nahmias NC. Cephalic vein cut-down verses percutaneous access: a retrospective study of complications of implantable venous access devices. American journal of surgery. 2006;192(1):63-7.

27. Matiotti-Neto M, Eskander MF, Tabatabaie O, Kasumova G, Bliss LA, Ng SC, et al. Percutaneous versus Cut-Down Technique for Indwelling Port Placement. The American surgeon. 2017;83(12):1336-42.

28. Ragusa M, Alberti D, Argento R, Avenia N, Bartolucci R, Esposito S, et al. [Central venous access systems in the oncologic patient]. Minerva chirurgica. 2000;55(3):139-46.

29. Rhu J, Jun KW, Song BJ, Sung K, Cho J. Cephalic vein approach for the implantable central venous access: A retrospective review of the single institution's experiences; Cohort Study. Medicine. 2019;98(46):e18007.

30. Otsubo R, Hatachi T, Shibata K, Yoshida T, Watanabe H, Oikawa M, et al. Evaluation of totally implantable central venous access devices with the cephalic vein cut-down approach: Usefulness of preoperative ultrasonography. Journal of surgical oncology. 2016;113(1):114-9.

31. Vetter N, Koscielny A, Schäfer N, Kalff JC, Standop J. Komplikationen und Funktionsdauer intravenöser Portkathetersysteme in Abhängigkeit des Zugangswegs. Gefässchirurgie. 2013;18(8):708-13.

32. Ayadi S, Ksantini R, Maghrebi H, Daghfous A, Ayadi M, Fteriche F, et al. [Totally implantable venous access ports by cephalic vein cut-down for patients receiving chemotherapy]. La Tunisie medicale. 2011;89(8-9):699-702.

33. Conessa C, Talfer S, Herve S, Chollet O, Poncet JL. [Cephalic vein access for implantable venous access devices. Technique and long-term follow-up]. Revue de laryngologie - otologie - rhinologie. 2002;123(3):143-8.

34. Di Carlo I, Cordio S, La Greca G, Privitera G, Russello D, Puleo S, et al. Totally implantable venous access devices implanted surgically: a retrospective study on early and late complications. Archives of surgery (Chicago, Ill : 1960). 2001;136(9):1050-3.

35. Hashimoto S, Otsubo R, Adachi M, Doi R, Shibata K, Sano I, et al. Cephalic Vein Cut-down for Totally Implantable Central Venous Access Devices With Preoperative Ultrasonography by Surgical Residents. In vivo (Athens, Greece). 2019;33(6):2079-85.

36. Hoareau-Gruchet F, Rtail R, Sulaj H, Khirnetkina A, Reyt E, Righini CA. [Complications after insertion of a totally implantable venous access port in patients treated with chemotherapy for head and neck squamous cell carcinoma]. Annales d'oto-laryngologie et de chirurgie cervico faciale : bulletin de la Societe d'oto-laryngologie des hopitaux de Paris. 2009;126(2):43-52.

37. Horng HC, Yuan CC, Chao KC, Cheng MH, Wang PH. A simple method to accurately position Port-A-Cath without the aid of intraoperative fluoroscopy or other localizing devices. Journal of surgical oncology. 2007;95(7):582-6.

38. Iorio O, Gazzanelli S, D'Ermo G, Pezzolla A, Gurrado A, Testini M, et al. A Prospective, Comparative Evaluation on Totally Implantable Venous Access Devices by External Jugular Vein versus Cephalic Vein Cutdown. The American surgeon. 2018;84(6):841-3.

39. Marcy PY, Magne N, Castadot P, Bailet C, Macchiavello JC, Namer M, et al. Radiological and surgical placement of port devices: a 4-year institutional analysis of procedure performance, quality of life and cost in breast cancer patients. Breast cancer research and treatment. 2005;92(1):61-7.

40. Povoski SP. A prospective analysis of the cephalic vein cutdown approach for chronic indwelling central venous access in 100 consecutive cancer patients. Annals of surgical oncology. 2000;7(7):496-502.

41. Repelaer van Driel OJ, Kuin CM, van de Velde CJ. Surgically implanted subcutaneous venous access devices in cancer patients. The Netherlands journal of surgery. 1988;40(4):97-9.

42. Sarzo G, Finco C, Parise P, Savastano S, Vecchiato M, Degregori S, et al. Insertion of prolonged venous access device: a comparison between surgical cutdown and percutaneous techniques. Chirurgia italiana. 2004;56(3):437-42.

43. Schumacher M, Wagner RH. Central venous port system associated thromboses: outcome in 3498 implantations and literature review. German medical science : GMS e-journal. 2007;5:Doc06.

44. Wenke K, Markewitz A. [Fully implantable catheter systems. Long-term results--complications]. Fortschritte der Medizin. 1990;108(14):276-9.

45. Adamus R, Beyer-Enke S, Otte P, Loose R. [Ultrasound-guided puncture of the subclavian vein to implant central venous ports]. RoFo : Fortschritte auf dem Gebiete der Rontgenstrahlen und der Nuklearmedizin. 2002;174(11):1450-3.

46. Apsner R, Muhm M, Unver B, Horl WH, Sunder-Plassmann G. Expanding our interventional skills: placement of totally implantable injection ports by internists/intensivists. Acta medica Austriaca. 2001;28(1):23-6.

47. Araujo C, Silva JP, Antunes P, Fernandes JM, Dias C, Pereira H, et al. A comparative study between two central veins for the introduction of totally implantable venous access devices in 1201 cancer patients. European journal of surgical oncology : the journal of the European Society of Surgical Oncology and the British Association of Surgical Oncology. 2008;34(2):222-6.

48. Aziret M, İrkörücü O, Gökler C, Reyhan E, Çetinkünar S, Çil T, et al. Performance of venous port catheter insertion by a general surgeon: a prospective study. Int Surg. 2015;100(5):827-35.

49. Bademler S, Ucuncu M, Yildirim I, Karanlik H. Risk factors for complications in cancer patients with totally implantable access ports: A retrospective study and review of the literature. The Journal of international medical research. 2019;47(2):702-9.

50. Ballarini C, Intra M, Pisani Ceretti A, Cordovana A, Pagani M, Farina G, et al. Complications of subcutaneous infusion port in the general oncology population. Oncology. 1999;56(2):97-102.

51. Barrios CH, Zuke JE, Blaes B, Hirsch JD, Lyss AP. Evaluation of an implantable venous access system in a general oncology population. Oncology. 1992;49(6):474-8.

52. Biffi R, de Braud F, Orsi F, Pozzi S, Mauri S, Goldhirsch A, et al. Totally implantable central venous access ports for long-term chemotherapy. A prospective study analyzing complications and costs of 333 devices with a minimum follow-up of 180 days. Annals of oncology : official journal of the European Society for Medical Oncology / ESMO. 1998;9(7):767-73.

53. Brooks AJ, Alfredson M, Pettigrew B, Morris DL. Ultrasound-guided insertion of subclavian venous access ports. Annals of the Royal College of Surgeons of England. 2005;87(1):25-7.

54. Campisi C, Assenza M, Zappala A, Di Paola M, Palazzo F, Di Muzio M, et al. Surgical technique and biomaterials for totally implanted port catheter systems. Journal of chemotherapy (Florence, Italy). 1997;9(2):155-6.

55. Capaccioli L, Nistri M, Distante V, Rontini M, Manetti A, Stecco A. [Insertion and management of long-term central venous devices: role of radiologic imaging techniques]. La Radiologia medica. 1998;96(4):369-74.

56. Chang DH, Boecker J, Hellmich M, Krug KB. [Experiences with ultrasound-guided port implantations via the lateral subclavian vein: a retrospective analysis of 1532 patients]. RoFo : Fortschritte auf dem Gebiete der Rontgenstrahlen und der Nuklearmedizin. 2012;184(8):726-33.

57. de Gregorio MA, Miguelena JM, Fernandez JA, de Gregorio C, Tres A, Alfonso ER. Subcutaneous ports in the radiology suite: an effective and safe procedure for care in cancer patients. European radiology. 1996;6(5):748-52.

58. Garajova I, Nepoti G, Paragona M, Brandi G, Biasco G. Port-a-Cath-related complications in 252 patients with solid tissue tumours and the first report of heparin-induced delayed hypersensitivity after Port-a-Cath heparinisation. European journal of cancer care. 2013;22(1):125-32.

59. Guner OS, Tumay LV, Zorluoglu A. [Subclavian venous port catheter implantation in general surgery: 238 cases from a single center]. Turkiye Klinikleri Journal of Medical Sciences. 2013;33:1330-6.

60. HEIBL C, TROMMET V, BURGSTALLER S, MAYRBAEURL B, BALDINGER C, KOPLMÜLLER R, et al. Complications associated with the use of Port-a-Caths in patients with malignant or haematological disease: a single-centre prospective analysis. European journal of cancer care. 2010;19(5):676-81.

61. Herrmann KA, Waggershauser T, Helmberger T, Heinemann V, Sittek H, Reiser M. [Percutaneous interventional radiologic implantation of intravenous port-catheter systems]. Der Radiologe. 1999;39(9):777-82.

62. Karanlik H, Kurul S. Modification of approach for totally implantable venous access device decreases rate of complications. Journal of surgical oncology. 2009;100(3):279-83.

63. Keum DY, Kim JB, Chae MC. Safety of a totally implantable central venous port system with percutaneous subclavian vein access. The Korean journal of thoracic and cardiovascular surgery. 2013;46(3):202-7.

64. Kluge A, Stroh H, Wagner D, Rauber K. [The fluoroscopy-guided implantation of subcutaneous venous ports: the complications and long-term results]. RoFo : Fortschritte auf dem Gebiete der Rontgenstrahlen und der Nuklearmedizin. 1998;169(1):63-7.

65. Koonings PP, Given FT, Jr. Long-term experience with a totally implanted catheter system in gynecologic oncologic patients. Journal of the American College of Surgeons. 1994;178(2):164-6.

66. Leclerc YE, Loutfi A. Implantable device for venous access. Canadian journal of surgery Journal canadien de chirurgie. 1987;30(2):127-9.

67. Leinung S, Wurl P, Anders K, Deckert F, Schonfelder M. [Port catheter fractures in 361 implanted port systems. Analysis of the causes--possible solutions--review of the literature]. Der Chirurg; Zeitschrift fur alle Gebiete der operativen Medizen. 2002;73(7):696-9.

68. Lokich JJ, Bothe A, Jr., Benotti P, Moore C. Complications and management of implanted venous access catheters. Journal of clinical oncology : official journal of the American Society of Clinical Oncology. 1985;3(5):710-7.

69. Lorch H, Zwaan M, Kagel C, Weiss HD. Central venous access ports placed by interventional radiologists: experience with 125 consecutive patients. Cardiovascular and interventional radiology. 2001;24(3):180-4.

70. Matsushima H, Adachi T, Iwata T, Hamada T, Moriuchi H, Yamashita M, et al. Analysis of the Outcomes in Central Venous Access Port Implantation Performed by Residents via the Internal Jugular Vein and Subclavian Vein. Journal of surgical education. 2017;74(3):443-9.

71. Miao J, Ji L, Lu J, Chen J. Randomized clinical trial comparing ultrasound-guided procedure with the Seldinger's technique for placement of implantable venous ports. Cell biochemistry and biophysics. 2014;70(1):559-63.

72. Nelson BE, Mayer AR, Tseng PC, Schwartz PE. Experience with the intravenous totally implanted port in patients with gynecologic malignancies. Gynecologic oncology. 1994;53(1):98-102.

73. Orsi F, Grasso RF, Arnaldi P, Bonifacio C, Biffi R, De Braud F, et al. Ultrasound guided versus direct vein puncture in central venous port placement. The journal of vascular access. 2000;1(2):73-7.

74. Ozyuvaci E, Kutlu F. Totally implantable venous access devices via subclavian vein: a retrospective study of 368 oncology patients. Advances in therapy. 2006;23(4):574-81.

75. Plumhans C, Mahnken AH, Ocklenburg C, Keil S, Behrendt FF, Gunther RW, et al. Jugular versus subclavian totally implantable access ports: catheter position, complications and intrainterventional pain perception. European journal of radiology. 2011;79(3):338-42.

76. Poorter RL, Lauw FN, Bemelman WA, Bakker PJ, Taat CW, Veenhof CH. Complications of an implantable venous access device (Port-a-Cath) during intermittent continuous infusion of chemotherapy. European journal of cancer (Oxford, England : 1990). 1996;32a(13):2262-6.

77. Shen-Gunther J, Mannel RS, Walker JL, Gold MA, Johnson GA. Outpatient implantation of a central venous access system in gynecologic oncology patients. The Journal of reproductive medicine. 2003;48(11):875-81.

78. Shetty PC, Mody MK, Kastan DJ, Sharma RP, Burke MW, Venugopal C, et al. Outcome of 350 implanted chest ports placed by interventional radiologists. Journal of vascular and interventional radiology : JVIR. 1997;8(6):991-5.

79. Shiono M, Takahashi S, Kakudo Y, Takahashi M, Shimodaira H, Kato S, et al. Upper arm central venous port implantation: a 6-year single institutional retrospective analysis and pictorial essay of procedures for insertion. PloS one. 2014;9(3):e91335.

80. Simpson KR, Hovsepian DM, Picus D. Interventional radiologic placement of chest wall ports: results and complications in 161 consecutive placements. Journal of vascular and interventional radiology : JVIR. 1997;8(2):189-95.

81. Suslu H, Arslan G, Tural K. [Venous port implantation in adult patients: retrospective evaluation]. Agri : Agri (Algoloji) Dernegi'nin Yayin organidir = The journal of the Turkish Society of Algology. 2012;24(1):32-6.

82. Tagliari AP, Staub FL, Guimaraes JR, Migliavacca A, Mossmann Dda F. Evaluation of three different techniques for insertion of totally implantable venous access device: A randomized clinical trial. Journal of surgical oncology. 2015;112(1):56-9.
