# Supplementary figures and images for: Meta-analysis of primary open versus closed cannulation strategy for totally implantable venous access port implantation
Source: Langenbecks Arch Surg. 2021 Jan 9;406(3):587–96. doi: 10.1007/s00423-020-02057-w (PMC8106576; doi:10.1007/s00423-020-02057-w)

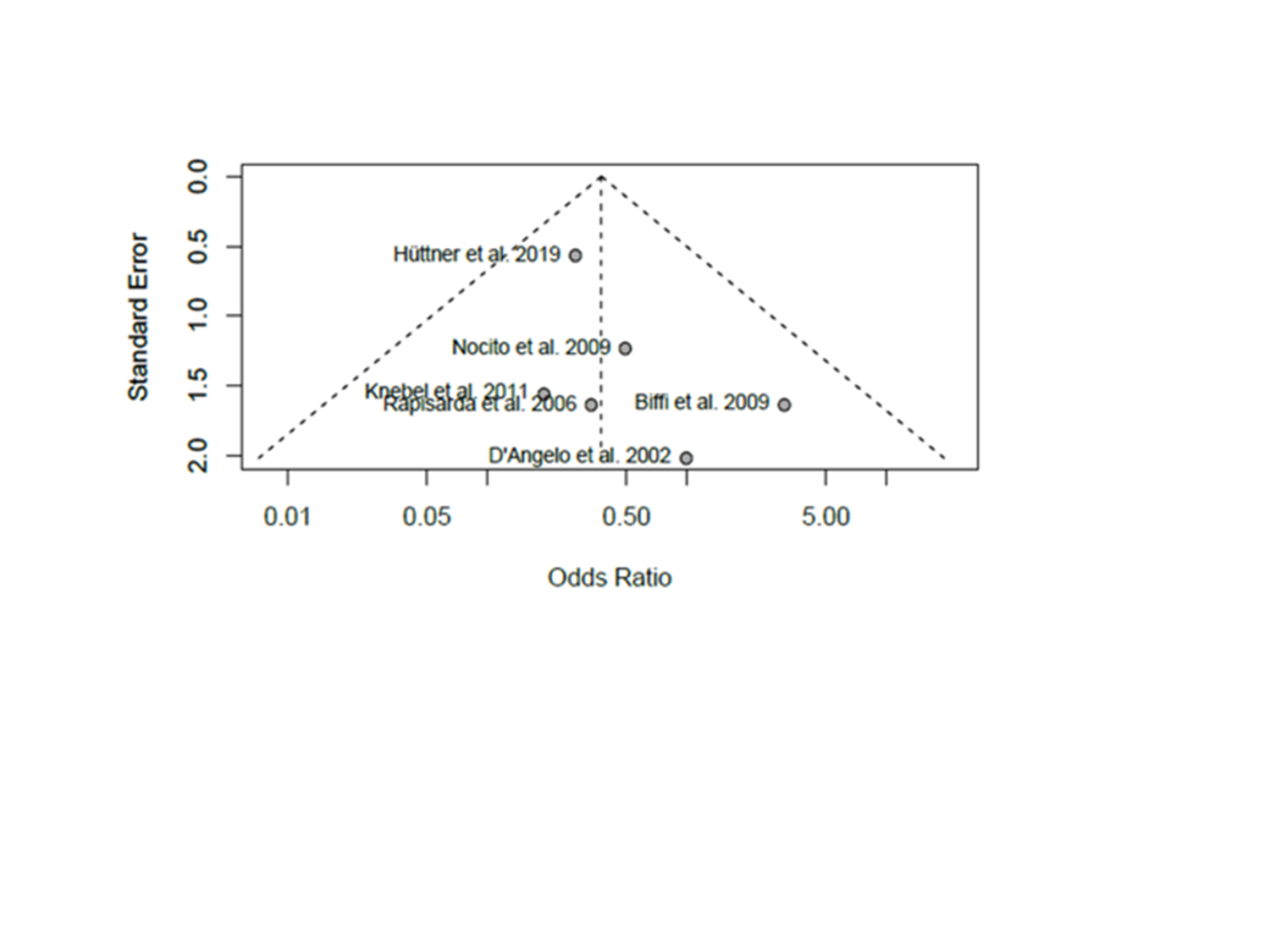

Supplement: Supplementary file 3 — Funnel plot for the endpoint pneumothorax (PNG 4948 kb) [file 423_2020_2057_Fig4_ESM.png]

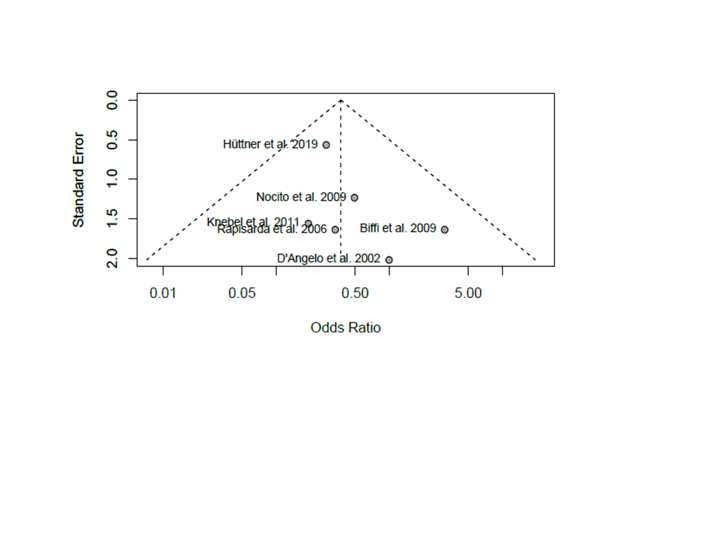

Supplement: Supplementary file 4 — High resolution (TIFF 1142 kb) [file 423_2020_2057_MOESM3_ESM.tiff]
